# Design and rationale of the pulse field ablation for postinfarction ventricular tachycardia: The ASCEND-VT pilot study

**DOI:** 10.1016/j.hroo.2026.04.008

**Published:** 2026-04-16

**Authors:** Jakub Sroubek, Bryan Baranowski, Mandeep Bhargava, Thomas D. Callahan, Mina K. Chung, Roy Chung, Thomas J. Dresing, Koji Higuchi, Ayman Hussein, Mohamed Kanj, Arshneel Kochar, Robert A. Koeth, Justin Z. Lee, David O. Martin, Walid I. Saliba, Tyler Taigen, Niraj Varma, Arwa Younis, Oussama M. Wazni, Pasquale Santangeli

**Affiliations:** Cardiac Pacing and Electrophysiology Section, Department of Cardiovascular Medicine, Cleveland Clinic, Cleveland, Ohio

**Keywords:** Ventricular tachycardia, VT ablation, Pulsed field ablation, Radiofrequency ablation, Ischemic cardiomyopathy

## Abstract

**Background:**

Ventricular tachycardia (VT) ablation in patients with ischemic cardiomyopathy (ICM) continues to be a challenging procedure with variable outcomes. Most current ablations are done using radiofrequency ablation (RFA). The new pulsed field ablation (PFA) technology may be able to achieve similar or superior procedural outcomes but potentially over shorter periods of time with less intravascular fluid infusion, but this possibility has never been tested.

**Objective:**

This study aimed to compare the procedural efficacy and safety of VT ablation in patients with ICM using focal PFA vs RFA catheters.

**Methods:**

This ASCEND-VT pilot study is a single-center trial randomizing patients to RFA vs PFA VT ablation in a 1:1 fashion. The new focal PFA catheter will be used under an investigational device exemption granted by the US Food and Drug Administration. Patients with mechanical valves will be excluded. The primary ablation target will be homogenization of endocardial late abnormal ventricular activity. Patients will be followed for 6 months after the procedure.

**Results:**

40 individuals will be randomized. The primary efficacy metric will be the ablation time needed to eliminate late abnormal ventricular activities in each patient. Additional efficacy and safety endpoints will be interrogated after the procedure and during outpatient follow-up.

**Conclusion:**

This is the first pilot randomized controlled trial comparing the safety and efficacy of focal PFA vs RFA VT ablation in ICM. Its results will inform the design of future larger studies that will further establish the clinical utility of PFA in the context of scar-related VT ablation.

**Key Findings**

▪ ASCEND-VT is a pilot trial that randomizes patients with ischemic cardiomyopathy and ventricular tachycardia to endocardial ablation using standard radiofrequency vs novel pulsed field ablation catheter.
▪ The intervention arm will use the new FARAPOINT focal pulsed field ablation catheter (under the US Food and Drug Administration’s investigational device exemption).
▪ The immediate procedural endpoint in all patients will be total elimination of abnormal electrograms, specifically late abnormal ventricular activity (LAVA).
▪ The trial’s primary efficacy metric is the time needed to eliminate LAVAs in the 2 study arms. This will be tested for noninferiority.
▪ Additional efficacy and safety metrics will be assessed after the ablation and over the course of a 6-month follow-up period.

## Introduction

Ventricular tachycardia (VT) in the setting of ischemic cardiomyopathy (ICM) is a potentially life-threatening arrhythmia with a high rate of recurrence.^1^^,^^2^ Although implantable cardioverter-defibrillator (ICD) therapy can effectively interrupt VT and improve mortality,^3^^,^^4^ ICDs alone are often insufficient as long-term monotherapy in patients given the effect of ICD shocks on mortality, heart failure, and patient quality of life.^5^, ^6^, ^7^ Additional therapies for VT include antiarrhythmic medications (which can be ineffective and put patients at risk of adverse effects) and catheter ablation.^2^^,^^8^ Indeed, radiofrequency ablation (RFA) of VT has been proven to be effective in multiple clinical studies.^1^^,^^2^^,^^8^, ^9^, ^10^, ^11^, ^12^

The field of VT catheter ablation has evolved dramatically over the past several decades. However, the modern approach to VT ablation in patients with ICM relies heavily on substrate ablation targeting abnormal local delayed/fragmented electrograms (late abnormal ventricular activity [LAVA]) within the confines of the scar.^13^, ^14^, ^15^, ^16^, ^17^, ^18^ Indeed, successful elimination of LAVAs has been associated with improved VT-free survival.^14^^,^^15^ Unfortunately, substrate ablation using RFA is not always straightforward. Because RFA lesion formation is often inconsistent in scarred myocardium,^19^ abnormal potentials cannot be completely eliminated in a substantial proportion of patients.^15^^,^^20^ Furthermore, RFA substrate homogenization is frequently a lengthy procedure necessitating prolonged exposure to anesthetic agents and injection of substantial amounts of intravenous fluid via the open-irrigated RFA catheters.

Pulsed field ablation (PFA) is primarily a nonthermal ablation technique that causes cell death by creating pores in myocyte membranes.^21^^,^^22^ Although the nominal dimensions of the myocardial PFA lesions may be comparable with RFA, the former may be more efficient and consistent in creating lesions in scarred substrate.^23^, ^24^, ^25^ Just as importantly, in contrast to RFA, PFA lesions can be created much more rapidly (typically <10 seconds per site) and with the use of minimal or no irrigation. In contrast, the use of PFA may be associated with an increased risk of certain adverse events that are relatively specific to this technology; as shown in ablation studies targeting atrial arrhythmias, this includes the risk of coronary vasospasm and hemolysis-related acute kidney injury.^26^^,^^27^ Even so, the recently published aggregate ADVANTAGE AF data suggest that the overall safety profile for atrial PFA ablations is very favorable (primary safety endpoint rate 2.0%; myocardial infarction rate 0.2%; and clinically relevant hemolysis rate 0.6%, although no events required hemodialysis).^28^

Several small studies and case reports have examined the safety and efficacy of PFA in the context of VT ablation.^29^, ^30^, ^31^, ^32^, ^33^, ^34^ Larger cohort data on PFA ablation of VT and premature ventricular contractions have only been published recently. This includes our own experience with a large-footprint pentaspline PFA catheter (n = 11)^35^ and a larger series by Ruwald et al^36^ (n = 35) that relied on a focal ablation catheter and PFA generator. The outcomes in VT ablation were similar to what was previously reported with focal RFA catheters. In particular, Ruwald et al^36^ saw a 55% 1-year recurrence rate in VT ablation patients. However, it is important to state that the studied population was enriched for high-complexity cases with intricate, nonischemic substrate. Although the complication rates in these (and other) studies are reassuring,^35^, ^36^, ^37^ the safety of PFA is yet to be confirmed in a more uniform population.

Therefore, we have designed a randomized pilot study intended to directly compare the safety and efficacy of PFA vs standard RFA using a prespecified procedural endpoint (elimination of abnormal potentials) in patients with ICM and VT. We hypothesize that the time needed to eliminate the abnormal potentials will be shorter with PFA than with RFA. We will use the new focal FARAPOINT PFA catheter that has similar dimensions and handling to the existing RFA catheters.

## Methods

The ASCEND-VT trial is a single-center, randomized noninferiority pilot study comparing the efficacy and safety of the novel FARAPOINT PFA catheter vs standard RFA catheters during ablation of VT in patients with ICM. The study conforms to the Declaration of Helsinki. It has been approved by the Cleveland Clinic Internal Review Board and the US Food and Drug Administration, which granted an investigational device exception (G220079). The trial is listed in the ClinicalTrials.gov registry (NCT06891456). The study is investigator initiated and represents collaborative research between Cleveland Clinic and Boston Scientific Corporation. It is funded by Boston Scientific Corporation. Given that this is a trial design manuscript, there are no primary data available for sharing.

### Trial design

A total of 40 patients (up to 50 patients will be screened) with ICM and VT will be randomized to PFA vs RFA for VT ablation in a 1:1 fashion using a block-randomization design (block size = 4). Follow-up will be 6 months. The total enrollment period is anticipated to be 12 months.

### Eligibility

Inclusion and exclusion criteria are presented in Table 1. Briefly, eligible patients must have a documented history of sustained monomorphic VT in the setting of ICM with a left ventricular ejection fraction (LVEF) of ≥20%. Important exclusion criteria include the presence of mechanical valves or a left ventricular (LV) assist device; patients with suspected epicardial substrate will also be excluded. Potential participants will be identified and approached in outpatient electrophysiology clinics or in the inpatient setting. After reviewing a study information sheet, discussing the study with the research coordinator, and having all their questions addressed, interested subjects will provide a written informed consent before enrolling in the study.

**Table 1:** Study inclusion and exclusion criteria

Inclusion criteria		Exclusion criteria
Age ≥18 y		Unable to provide an informed consent
Ischemic heart disease with previous myocardial infarction		Idiopathic VT
LVEF ≥20% estimated by TTE within 90 d before enrollment		Mobile LV thrombus
Documented sustained monomorphic VT with any of the following characteristics:		Acute coronary syndrome within the preceding 2 mo (if incessant VT ≥1 mo before enrollment)
	≥2 documented episodes in patients with ICD	Comorbidity likely to limit survival to <12 mo
	≥1 documented episode in patients without ICD	New York Heart Association class IV heart failure
	Recurrent VT despite antiarrhythmic medications (ineffective, contraindicated, or not tolerated) or ICD interventions	Estimated glomerular filtration rate <30 mL/min/1.73 m^2^
Provision of a signed and dated informed consent form		Thrombocytopenia or coagulopathy
Stated willingness to comply with all study procedures and availability for the duration of the study		Contraindication to heparin
		Pregnancy or lactation
		Cardiac surgery within the past 2 mo
		Active infection
		Clinical, laboratory, or imaging evidence of active ischemia
		Severe left heart valvular heart disease (aortic/mitral stenosis or regurgitation)
		Any concomitant congenital heart disease
		Previous catheter or surgical ablation of VT within the past 2 mo
		Anticipated need for epicardial mapping and ablation
		For individuals with no preexisting ICD: ineligibility for an ICD implant
		Preexisting LVAD or other hemodynamic assist device
		Present mechanical heart valve
		Cardiogenic shock unless it is caused by incessant VT

ICD = implantable cardioverter-defibrillator; LV = left ventricle; LVAD = left ventricular assist device; LVEF = left ventricular ejection fraction; MI = myocardial infarction; TTE = transthoracic echocardiogram; VT = ventricular tachycardia.

**Figure 1:**
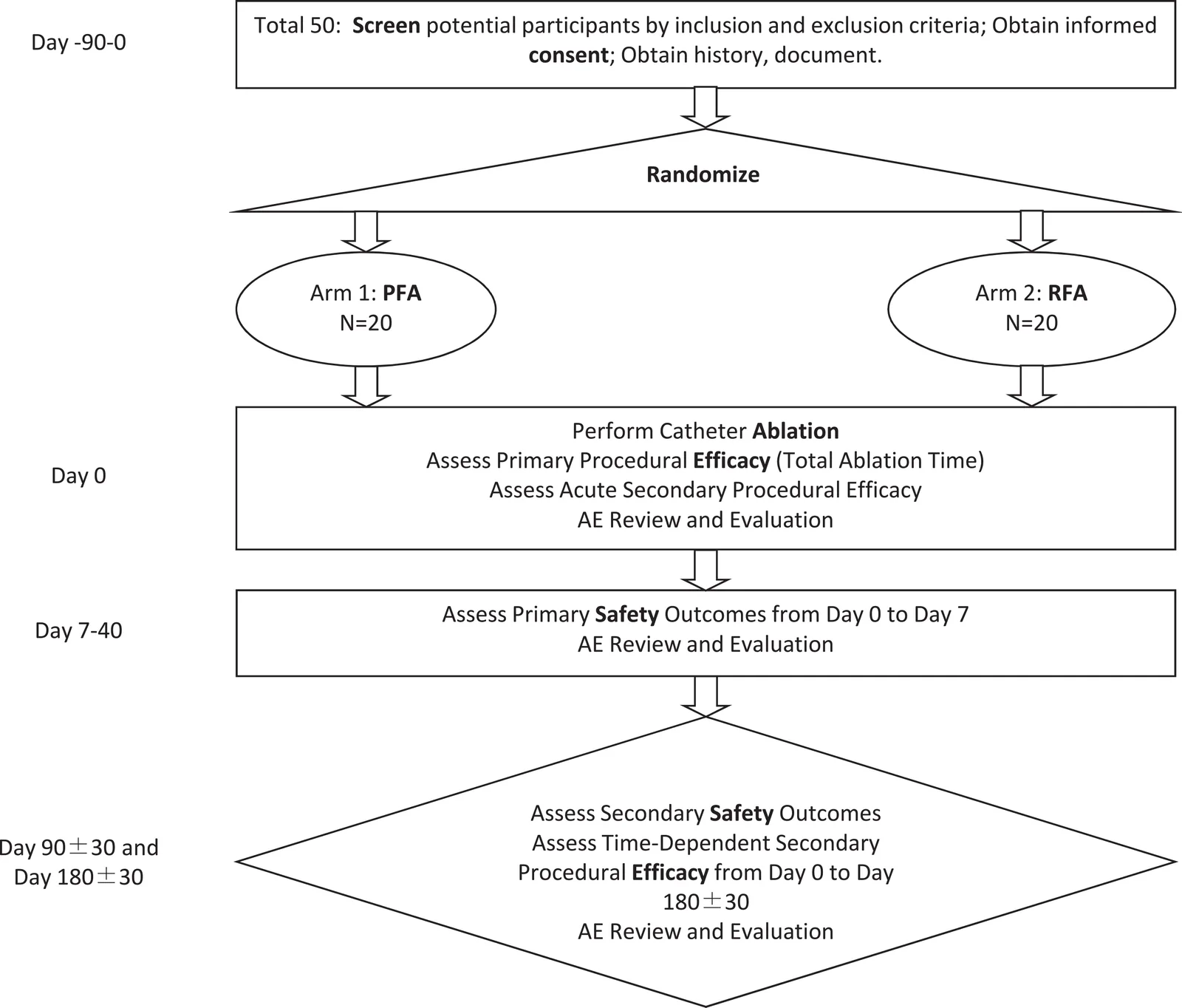
ASCEND-VT trial flowchart. AE = adverse event; PFA = pulsed field ablation; RFA = radiofrequency ablation.

The study protocol and follow-up plan are presented in Figure 1.

### Baseline data collection

During the baseline office visit (or inpatient interview), data will be collected including patient medical history, demographics, and medication regimen. In particular, this will include physical and cardiac assessment, cardiovascular disease history, cerebrovascular disease history, cardiac procedure history, atrial and ventricular arrhythmia history, subject comorbidities, pandemic history, and information on antiarrhythmic (class I and III) and anticoagulation medications.

Further testing such as electrocardiogram and echocardiogram will be performed. The 36-item short form health survey will be administered.

Device interrogation will be performed, and information including the description of the last 10 VT episodes and resulting treatment (rate, duration, antitachycardia pacing/shock before enrollment, programming of therapy zones, etc) will be collected.

### Ablation procedure: General considerations

In both study arms, the ablation procedure will be done under general anesthesia or conscious sedation (with local anesthesia administered to the vascular access sites), per individual operator’s preference. Antithrombotic agents will be continued or held before the procedure at the primary operator’s discretion. Femoral intravascular access will be obtained under ultrasound guidance. Intracardiac echocardiography and a quadripolar right ventricular pacing catheter will be used in all cases. A multipolar mapping catheter and an ablation catheter (see below) will be advanced into the LV via transseptal or retroaortic approach, as deemed appropriate by the operator.

### Ablation procedure: Baseline inducibility

Baseline programmed ventricular stimulation (PVS) will be performed at the start of the procedure. This will entail using up to 3 extrastimuli down to the ventricular effective refractory period or coupling intervals of 200 ms (whichever occurs sooner) or until VT or ventricular fibrillation is induced. The use of multiple drive train cycle lengths (eg, 600 and 400 ms) and at least 2 pacing sites (eg, right ventricle and LV) is encouraged.

Any induced sustained monomorphic VT will be recorded; activation mapping of hemodynamically stable VT is permissible at this point, but ablation should not be performed. Sustained VT should be terminated using overdrive pacing or external cardioversion.

### Ablation procedure: Substrate mapping

In the control arm, the choice of mapping system and mapping catheters will depend on the operator’s discretion using a commercially available electroanatomic mapping system and mapping catheter. In the intervention arm, mapping will be done using the OPAL HDx mapping system (Boston Scientific, Marlborough, MA) along with the ORION multipolar basket catheter (Boston Scientific). Ablation catheters (outlined below) may also be used for mapping in all patients.

In both study arms, substrate mapping will be done during sinus rhythm and/or paced rhythm.

### Ablation procedure: Definition of substrate target

The main substrate ablation target will be LAVA electrograms within the abnormal substrate. These are defined as sharp high-frequency ventricular potentials, possibly of low amplitude, distinct from the far-field ventricular electrogram occurring any time during or after the far-field ventricular electrogram in sinus/paced rhythm that sometimes display fractionation or double or multiple components separated by very-low-amplitude signals or an isoelectric interval and are poorly coupled to the rest of the myocardium.^15^ During substrate mapping, all sites with LAVAs will be marked using the mapping system. In addition, in patients with stable VTs, activation and entrainment mapping will be permitted to prioritize LAVA areas to target, whereas, in patients with unstable VTs, pace mapping from substrate areas with LAVAs will also be permitted.

### Ablation procedure: Substrate ablation

All LAVA areas will be targeted for ablation unless concern for collateral damage is thought to outweigh the benefits by the operator (eg, vicinity of conduction system). Ablation should not be performed in the proximity of metallic devices (such as intracardiac lead electrodes) to avoid arcing, cardiac trauma, and/or damage to the devices.

In the control arm, ablation will be done using commercially available contemporary RFA contact force–sensing slow-flow irrigated RFA catheters using standard irrigation settings and 30–50 W output for at least 30 seconds. The goal of each lesion will be the complete elimination of LAVA and/or local myocardial noncapture during high-output pacing (10 mA/2 ms to 20 mA/2 ms).

In the intervention arm, PFA will be done via the FARAPOINT focal catheter (Boston Scientific) using 5 bipolar/biphasic trains of 2.0 kV applications delivered over 2.5 seconds; the number of applications will be repeated based on lesion depth needs. There will be no limit on the number of RFA or PFA lesions.

In both study arms, the ablated areas will be remapped using the same catheter used for baseline substrate mapping and elimination of LAVAs will be confirmed by the primary operator (post hoc data review by the principal investigators [P.S. and J.S.] to confirm LAVA elimination will also be performed, and any discrepancies will be documented). In addition, high-output pacing (10 mA/2 ms to 20 mA/2 ms) will be delivered to the ablated sites to confirm noncapture at high or very high output. If persistent LAVAs and/or locations with persistent myocardial capture are identified, additional ablation is encouraged in both study arms.

Patients with an anticipated need for epicardial access will be excluded from the study. However, in cases where ad hoc epicardial access becomes necessary in an enrolled study subject (eg, unexpected identification of an epicardial VT focus during the procedure), epicardial mapping/ablation can be performed as clinically necessary; however, only RFA ablation is permitted in the pericardial space (or inside the coronary sinus system).

### Ablation procedure: Subsequent inducibility

PVS will be repeated in hemodynamically stable patients using the same protocol as before (when testing for baseline inducibility). Mapping and ablation of any sustained VT (thought to be clinically relevant and/or exhibiting tachycardia cycle length of ≥240 ms) will be encouraged using any strategy deemed suitable by the operator. If clinically appropriate, PVS will then be repeated until no further sustained VT (meeting the criteria mentioned earlier) is inducible. Cross-over to the other study arm at this point will be considered a protocol deviation.

### Postprocedural care

All study subjects will receive standard postprocedural care that will include observation for at least 1 night. Most medical management decisions, including the use of antithrombotic agents, will be left up to the discretion of the primary operator. At most 1 antiarrhythmic drug can be used for up to 1 month after ablation when it will be discontinued. ICD programming in the individual patient should be evaluated and verified after the procedure to allow for similar detection and treatment of VT as before the procedure. ICD interrogation will also include a review of lead parameters, and any deviation from preablation values will be recorded.

### Follow-up

The outline of the follow-up data collection strategy is presented in Table 2. Primary and procedural efficacy along with adverse event review will be performed immediately after the procedure and at the time of hospital discharge. Study visits 1 (7-40 days after the procedure) and 2 (90 ± 30 days after the procedure) will include functional assessment, ICD interrogation, and adverse event review. The final study visit (day 180 ± 30 after the procedure) will be a comprehensive assessment that will include transthoracic echocardiography and the 36-item short form health survey.

**Table 2:** Schedule of activities

Procedures	Screening and enrollment/baseline, day −90 to −1	Ablation procedure	Discharge	Study visit 1, day 7–40∗	Study visit 2, day 90 ± 30∗	Final study visit, day 180 ± 30
Informed consent	X					
Demographics	X					
Medical history	X					
Physical examination	X		X			X
NYHA assessment	X			X	X	X
Vital signs	X		X			X
TTE†	X					X
ECG	X					X
ICD interrogation‡	X	X		X	X	X
SF-36	X					X
Adverse event review and evaluation		X	X	X	X	X
Complete case report forms	X	X	X	X	X	X

ECG = electrocardiogram; ICD = implantable cardioverter-defibrillator; NYHA = New York Heart Association; SF-36 = 36-item short form health survey; TTE = transthoracic echocardiogram.

∗ In-office visit or telephone/virtual visit. ICD interrogation can be monitored remotely.

† TTE at baseline to be performed within 6 months of enrollment.

‡ ICD interrogation can be performed via remote monitoring.

### Endpoints

The efficacy and safety endpoints are presented in Table 3. In brief, the primary efficacy endpoint is the total time needed to ablate and eliminate all targeted LAVAs. The secondary efficacy endpoints include other procedural metrics (including postprocedural inducibility, total procedural duration, and fluid balance) and time-dependent outcome metrics such as total mortality, hospital admissions for cardiovascular indication, and recurrences of VT/ventricular fibrillation. Safety endpoints include a range of possible periprocedural complications, with particular attention paid to issues that may be more relevant to PFA; this includes any coronary events (including coronary vasospasm), acute kidney injury, and changes in LVEF and/or evidence of new wall motion abnormalities.

**Table 3:** Efficacy and safety endpoints

Primary efficacy endpoint	Objective/rationale
Time needed to eliminate LAVAs	This is the primary procedural efficacy metric highlighting a key potential advantage of PFA over RFA technology.
Acute secondary efficacy endpoints	Objective/rationale
Total procedural duration	These are additional procedural efficacy metrics expected to mirror the primary efficacy endpoint.
Total fluid administered by the ablation catheter	
Total fluid administered during the procedure	
Fluid balance at the end of the procedure	
Ongoing presence of LAVAs at the end of the procedure	A different efficacy metric. LAVA elimination may not always be possible with RFA and is likely always feasible with PFA, although the durability of this elimination remains to be determined.
VT noninducibility at the end of the procedure	A standard acute endpoint metric, although its utility has lately been challenged^45^
Time-dependent secondary efficacy endpoints (assessed 6 mo postoperatively)	Objective/rationale
Recurrences of sustained VT leading to appropriate ICD interventions (ATP or shock) or any symptomatic sustained VT	Standard clinical follow-up endpoints. The ASCEND-VT trial is not powered to show significant differences in these outcomes. However, if major differences are ultimately observed in these metrics, future studies will have to elucidate whether the procedural workflow of PFA needs to be adjusted.
Any documented sustained VT below the detection rate of the ICD	
All-cause mortality	
VT burden (number of appropriate ICD therapies in the 6 mo before ablation vs 6 mo after the ablation)	
Hospital admissions for cardiovascular causes	
Primary safety endpoints (assessed ≤7 d postoperatively)	Objective/rationale
Cardiac perforation	Benchmark safety data from the ThermoCool VT trial^10^ were based on similar endpoints.
Pericardial effusion with hemodynamic compromise or needing intervention	
New complete heart block	
Coronary spasm	
Acute MI	
New acute severe mitral regurgitation or aortic regurgitation	
Deep venous thrombosis	
Vascular injury requiring surgical or percutaneous intervention	
Pulmonary embolism	
Stroke	
Acute kidney injury (>0.5 mg/dL increase in creatinine from baseline within 24 h after the procedure)	
Death	
Secondary safety endpoints (assessed during follow-up)	Objective/rationale
Device- or procedure-related SAEs (the proportion of subjects in each treatment arm with at least 1 device- or procedure-related SAE)	Summary metric of SAE frequency
Acute kidney injury	Metrics of particular relevance to the PFA technology
Coronary vasospasm at any point during follow-up	
Changes in LVEF and/or new wall motion abnormalities at follow-up TTE (180 d)	

ATP = antitachycardia pacing; ICD = implantable cardioverter-defibrillator; MI = myocardial infarction; LAVA = late abnormal ventricular activity; LVEF = left ventricular ejection fraction; PFA = pulsed field ablation; RFA = radiofrequency ablation; SAE = serious adverse event; TTE = transthoracic echocardiogram; VT = ventricular tachycardia.

### Statistical analysis and power calculations

The primary null hypothesis is that the total ablation time needed for complete LAVA elimination (ie, primary efficacy metric) will not be different between the 2 study arms. This hypothesis will be tested using a 2-sample *t* test.

PFA lesion delivery time will be, by design, shorter (3 2.5-second pulse trains = 7.5 seconds per ablation site) than RFA (at least 30 seconds per lesion). Furthermore, effective RFA lesion dimensions may vary widely depending on the distribution of myocardial scar, whereas PFA lesions are expected to be more consistent.^25^^,^^38^ Therefore, we envisage the time needed to eliminate targeted LAVAs to differ by at least 50% between the 2 study arms. With a desired power of 80% and a significance level of 0.05, the predicted minimum enrollment is 36 patients (18 in each study arm). This will be increased to 40 patients to allow for a 10% withdrawal rate. 10 additional patients will be screened to allow for possible screen failures.

The secondary efficacy and both primary and secondary safety endpoints will be analyzed using *t* test, rank sum test, or Fisher’s exact test, as appropriate. Time-dependent outcomes will be analyzed as landmark events. However, if the event rates and/or dropout rates are high in either group (≥20%), time-to-event methodology will be adopted (Cox regression and Fine-Gray competing risk models, as appropriate). Univariate and multivariable models will be used.

## Discussion

The ASCEND-VT pilot trial is the first randomized prospective study designed to assess the procedural efficacy and safety of VT ablation in patients with ICM, using a focal PFA catheter vs standard RFA technology. The primary efficacy metric of ASCEND-VT is intended to directly compare PFA vs RFA with respect to the time that it takes to eliminate abnormal endocardial potentials (specifically, LAVAs) in this population.

This study design was chosen to test the anticipated primary advantage of the PFA technology in this context: its expected ability to achieve the same procedural endpoint as standard ablation but in a more streamlined fashion. If the ASCEND-VT study reveals similar ablation times between PFA and RFA along with comparable safety profiles, then that alone may boost the former technology’s clinical appeal given that the PFA apparatus does not require large volumes of open irrigation. Furthermore, if the ASCEND-VT study reveals a large primary effect size (ie, large difference in ablation times between the 2 study arms), then we may even postulate a trend toward procedural “superiority” of PFA, that is, substantially shorter ablation that can translate into shorter procedural times, which will confer additional benefits on the patients such as shorter exposure to anesthetic agents, less time under positive pressure ventilation, and overall lower procedural complication risk, as previously reported.^39^

With that in mind, we expect most of the other secondary outcome metrics (such as fluid input/balance and total procedural duration) to mirror the primary outcome. In further secondary analyses, the ASCEND-VT study will also compare the numbers of cases where total elimination of LAVAs could not be achieved. As discussed earlier, this endpoint is sometimes unachievable using RFA.^15^^,^^20^ In contrast, interpretation of this finding may be challenging considering the reversible nature of PFA in certain scenarios.^40^

The ASCEND-VT study will also look at hard clinical outcomes such as VT recurrences and all-cause mortality, although the power to detect any signal in this realm may be limited. Indeed, establishing overall clinical noninferiority of 1 ablation technology over another, for example, proving that the use of PFA results in lower VT recurrence or lower mortality than RFA, will necessitate a large-scale multicenter trial with an extended follow-up time. However, ASCEND-VT will provide important early information to design a large head-to-head comparative trial.

The ASCEND-VT study is also intended to systemically assess the safety of PFA in ICM VT ablations. Although the rate of vascular or major mechanical cardiac adverse events (eg, pericardial effusion and tamponade) is expected to be comparable between the 2 study arms, certain potential complications are relatively specific to PFA and will need to be separately assessed. First, early PFA literature (particularly in the context of atrial ablation) has shown that transient hemolysis is a relatively frequent consequence of PFA ablation, although the incidence of resulting end-organ damage is very low.^28^^,^^41^^,^^42^ For this reason, the ASCEND-VT trial will specifically track the rate of acute kidney injury after the procedure. Second, PFA in the atrium may result in vasospasm when applied in the vicinity of coronary arteries^26^^,^^43^; although patients in ASCEND-VT will only be subjected to ventricular endocardial PFA applications (ie, not near the epicardial coronary vessels), the possibility of vasospasm with or without substantial myocardial injury will be closely tracked. However, the potential for vasospasm is not unique to PFA alone, having previously been demonstrated with RFA, and will be evaluated in both arms. Third, even in the absence of new coronary injury, there is a theoretical concern that the uniform and large PFA lesions may have an effect on myocardial contractility, especially if applied outside of noncontractile scar^44^; therefore, the ASCEND-VT trial will pay particular attention to any perturbations in LVEF and/or evidence of new wall motion abnormalities after ablation.

The limitations of the ASCEND-VT trial primarily stem from the study’s relatively small size. As discussed earlier, the trial is not powered to detect differences in clinical outcomes (such as VT recurrences or mortality) between the 2 study arms. Similarly, the study may not be able to estimate the relative risk of PFA for presumably uncommon complications (eg, stroke). Furthermore, the trial is not designed to confirm the durability of LAVA elimination; this may be particularly relevant in the PFA arm where local electrogram elimination may be reversible in some cases. In addition, the ASCEND-VT trial will use different mapping systems and mapping catheters in each arm—a logistical constraint stemming from each mapping system’s compatibility with the used ablation catheters. This heterogeneity in the mapping apparatus may inadvertently result in the designation of slightly different ablation targets in each study arm. An analysis will be undertaken wherein the time needed to eliminate LAVA will be normalized to the total surface area exhibiting LAVA at baseline. This additional analysis would generate results independent of the used mapping system and may be a more objective metric of substrate ablation efficiency.

Although the primary study endpoint is elimination of all LAVAs, induction and mapping of VTs when feasible are also allowed to ensure targeting more rare subtypes of non–scar-related VTs. Given the randomized nature of this study, this would not be expected to substantially affect the primary study endpoint.

## Conclusion

The ASCEND-VT pilot study is a single-center, randomized trial designed to assess the procedural efficacy and safety of focal PFA vs standard RFA in patients with VT and ICM. If this study establishes noninferiority of the PFA technology in this regard, this result will inform the design of future multicenter studies powered to look at the overall clinical efficacy of PFA for VT ablation.

## Disclosures

Dr Sroubek has received speaking fees from Abbott and Biosense Webster and receives research grant support from Abbott. Dr Callahan has served as a consultant for Abbott, Boston Scientific, Medtronic, Philips, and ShockWave. Drs Higuchi and Lee have served as consultants for Abbott. Dr Hussein receives research grant support from Boston Scientific. Dr Kanj has received speaking honorarium from Boston Scientific and has served as a consultant for Boston Scientific. Dr Martin has served as a consultant for Medtronic. Dr Saliba has served as a consultant for Biosense Webster and Boston Scientific and as an advisory board member for Abbott, Biosense Webster, Boston Scientific, and Philips. Dr Taigen has served as a consultant for Biosense Webster and Medtronic. Dr Varma has served as a consultant for Abbott, Biotronik, Boston Scientific, and Medtronic. Drs Wazni and Santangeli have served as consultants for Abbott, Biosense Webster, Boston Scientific, and Medtronic. All other authors have reported that they have no relationships relevant to the contents of this paper to disclose.
